# Digital Transformation of Rheumatology Care in Germany: Cross-Sectional National Survey

**DOI:** 10.2196/52601

**Published:** 2025-01-06

**Authors:** Susann May, Robert Darkow, Johannes Knitza, Katharina Boy, Philipp Klemm, Martin Heinze, Nicolas Vuillerme, Pascal Petit, Patricia Steffens-Korbanka, Heike Kladny, Johannes Hornig, Peer Aries, Martin Welcker, Felix Muehlensiepen

**Affiliations:** 1 Center for Health Services Research Faculty of Health Sciences Brandenburg Brandenburg Medical School Theodor Fontane Rüdersdorf bei Berlin Germany; 2 FH JOANNEUM Gesellschaft mbH Graz Austria; 3 AGEIS Université Grenoble Alpes Grenoble France; 4 Institute for Digital Medicine University Hospital of Giessen and Marburg Philipps University Marburg Marburg Germany; 5 Department of Rheumatology, Immunology, Osteology Kerckhoff-Klinik GmbH Bad Nauheim Germany; 6 University Clinic for Psychiatry and Psychotherapy Brandenburg Medical School Immanuel Hospital Rüdersdorf Rüdersdorf bei Berlin Germany; 7 Institut Universitaire de France Paris France; 8 LabCom Telecom4Health Orange Labs & Univ. Grenoble Alpes CNRS, Inria, Grenoble INP-UGA Grenoble France; 9 Rheumapraxis an der Hase Osnabrück Germany; 10 MVZ für Rheumatologie Dr. Martin Welcker GmbH Planegg Germany; 11 Immunologikum Hamburg, Rheumatologie & Klinische Immunologie Hamburg Germany

**Keywords:** telemedicine, digital health technologies, rheumatology, COVID-19, questionnaire, telehealth, eHealth, digital health, survey, rheumatism, Germany, Europe, national, use, experience, attitude, opinion, perception, perspective, acceptance, preference, correlation

## Abstract

**Background:**

In recent years, health care has undergone a rapid and unprecedented digital transformation. In many fields of specialty care, such as rheumatology, this shift is driven by the growing number of patients and limited resources, leading to increased use of digital health technologies (DHTs) to maintain high-quality clinical care. Previous studies examined user acceptance of individual DHTs in rheumatology, such as telemedicine, video consultations, and mHealth. However, it is essential to conduct cross-technology and continuous analyses of user acceptance and DHT use to maximize the benefits for all relevant stakeholders.

**Objective:**

This study aimed to explore the current acceptance, use, and preferences regarding DHTs among patients in rheumatology care in Germany.

**Methods:**

Rheumatology patients from 3 clinics in Germany were surveyed to understand their perspectives on DHTs. The survey included main themes, including acceptance, preferences, COVID-19’s impact, potential, and barriers related to DHTs. The data were analyzed using descriptive statistics and correlation analysis.

**Results:**

Out of 337 participants, 53% (179/337) reported using DHTs. Specific technologies included wearables (72/337, 21%), mHealth apps (71/337, 21%), digital therapeutics (32/337, 9%), electronic prescriptions (30/337, 9%), video consultations (15/337, 4%), and at-home blood self-sampling (3/337, 1%). Nearly two-thirds (220/337, 65%) found DHTs useful, and 69% (233/337) held a generally positive attitude toward DHTs. Attitudes shifted positively during the COVID-19 pandemic for 40% (135/337) of participants. Higher education was more prevalent among DHT users (114/179, 63.7%) compared with nonusers (42/151, 27.8%; *P*=.02). The main potential benefits identified were location-independent use (244/337, 72%) and time-independent use (216/337, 64%). Key barriers included insufficient user knowledge (165/337, 49%) and limited information on DHTs (134/337, 40%).

**Conclusions:**

Patient acceptance and use of DHTs in rheumatology is increasing in Germany. A prospective, standardized monitoring of digital transformation in rheumatology care is highly needed.

## Introduction

Health care is undergoing a multidisciplinary digital transformation, which refers to “a process that aims to improve an entity by triggering significant changes to its properties through combinations of information, computing, communication, and connectivity technologies” [1].

Within the realm of rheumatology care, a notable surge in digital technologies has transpired in recent years [2,3]. This occurrence stems from the existing global disparity between the increasing number of patients with rheumatic and musculoskeletal disease (RMD) [4] and the static or even decreasing availability of human resources in the field of rheumatology care [5,6].

Consequently, Miloslavsky and Bolster [7] have discerned the amplified use of telemedicine as a prospective remedy to ensure the continuity of rheumatological care in the future [7]. Concurrently, and accelerated by the COVID-19 pandemic, the European Alliance of Associations for Rheumatology (EULAR) has recently published points to consider for remote care in RMD [8].

In Germany, a country struggling with a shrinking and aging population and a shortage of health care professionals, digital health technologies (DHTs) in rheumatology care have proven effective in improving rheumatology care [2,9]. Prominent among the prevailing DHTs are video consultations, sensors, wearables, digital health applications, and digital therapeutics with varied objectives (symptom checkers, ePRO [electronic patient-reported outcome] documentation, or patient education), social media platforms or messenger platforms [10], which can also be combined with home-based self-sampling [11-13].

Past studies have assessed patients’ and physicians’ acceptance of DHTs in the domain of rheumatology within Germany. These investigations primarily centered on individual digital modalities, such as telemedicine [13,14], mHealth [15], and video consultations [16,17]. Following the onset of the COVID-19 pandemic, the influence of this global crisis on the adoption of DHTs was also scrutinized [3,17]. It remains essential to continuously analyze the digital transformation in rheumatology to maximize the benefits for all relevant stakeholders. In particular, the patient perspective is critical to ensuring that DHTs actually improve health care, meets patient needs, and builds patient trust in the use of DHTs. Thus, the aim of this study was to explore the current acceptance, use, and preferences regarding DHTs among patients in rheumatology care in Germany.

## Methods

### Overview

To explore the patients’ perspectives and preferences regarding digital health systems in rheumatology care, we recruited rheumatology patients in 3 outpatient clinics. Inclusion criteria were being aged ≥18 years, literate in German, having the physical and mental ability to fill out a paper-pencil questionnaire, and having a diagnosis of RMD. The survey was conducted between February 2023 and April 2023. The 3 clinics were sent 125 questionnaires with the request to distribute the questionnaires to eligible patients.

The questionnaire was created based on a literature review and the results of a qualitative study with health care providers (HCPs) and nurses. The questionnaire addressed the following topics: acceptance of DHTs, use of DHTs, preferences for DHTs, the impact of COVID-19 on digital transformation, potential benefits, and barriers regarding DHTs, and sociodemographic characteristics. The survey was pilot-tested with 10 patients to detect necessary formatting and wording changes. Minor revisions were made accordingly. Please refer to Multimedia Appendix 1 for the German version of the questionnaire and Multimedia Appendix 2 for the translated version in English.

Data were analyzed using quantitative descriptive analysis and correlation analysis supported by SPSS (version 23.0; IBM). For correlation analyses, the data were converted into scores, and the Spearman rank correlation coefficient was calculated. The α error distribution was chosen to be 2-sided, as an undirected correlation is assumed to be open to hypotheses. These correlations had a correlation coefficient higher than 0.29.

### Ethical Considerations

This study was approved by the ethics committee of Brandenburg Medical School (E-02-20211028). All patients provided informed consent. The rheumatology clinics received an expense allowance of €150 (US $ 158) for the distribution and return of the questionnaires. The study data are anonymous. The questionnaires were digitized and then stored at the Center for Mental Health at Brandenburg Medical School.

## Results

### Patient Characteristics

In total, 337 patients with RMD completed the questionnaire. The number of patients rejecting participation was not measured. The study sample’s demographics are shown in Table 1. The mean age was 52.5 (SD 14.2) years; 64% (219/337) were female, 32% (108/337) were male, 1% were (3/337) nonbinary, and 2% (7/337) did not give any information on gender. On average, participants had been receiving rheumatologic treatment for a median 8 years (mean 9.35 years, SD 7.63; range: 1 month-399 months), and the median time with the diagnosis was 8.9 years (mean 10.2 years, SD 8.33; range: 3 months-579 months).

**Table 1:** Characteristics of participants.

Characteristics		Participants (N=337), n (%)
**Sex**		
	Female	219 (65)
	Male	108 (32)
	Nonbinary	3 (1)
	No data	7 (2)
**Diagnosis**		
	Axial spondyloarthritis	13 (4)
	Fibromyalgia	7 (2)
	Psoriatic arthritis	40 (12)
	Rheumatoid arthritis	182 (54)
	Sjörgren syndrome	10 (3)
	Spondyloarthritis	21 (6)
	Systemic lupus erythematosus	9 (3)
	Other	39 (12)
	No data	16 (5)
**Education level**		
	>10 years of school completed	156 (46)
	9-10 years of school completed	163 (48)
	Student	2 (1)
	No formal schooling	2 (1)
	No data	14 (4)
**Vocational training**		
	Apprenticeship	188 (56)
	Bachelor’s, master’s, or magister’s degree, diploma	109 (3)
	Started an apprenticeship	6 (2)
	Without apprenticeship	11 (3)
	No data	23 (7)
**Place of residence (number of inhabitants)**		
	Rural region (<5000)	70 (21)
	Small city (>5000-20,000)	73 (22)
	Medium city (>20,000-100,000)	63 (19)
	Large city (>100,000-1,000,000)	28 (8)
	Metropolis city (>1,000,000)	93 (28)
	No data	10 (3)

### Attitudes Toward DHTs

About half of the patients used DHTs overall (179/337, 53%) (Table 2). Overall, most patients (220/337, 65%) rated DHTs as useful, while a third remained neutral in their assessment of usefulness. Almost two-thirds of the patients (233/337, 69%) were positive or rather positive toward DHTs. Among all participants, 55% (185/377) stated that their attitude toward DHTs had not changed due to the COVID-19 pandemic. However, 40% (135/337) mentioned that their attitude had become more positive.

**Table 2:** Attitudes toward digital health technologies.

Items		Participants (N=337), n (%)
**Do you use digital health technologies?**		
	Yes	179 (53)
	No	151 (45)
	No data	7 (2)
**Digital health technologies are useful.**		
	Strongly agree	99 (29)
	Agree	121 (36)
	Neutral	99 (29)
	Disagree	5 (1)
	Strongly disagree	7 (2)
	No data	6 (2)
**How do you rate your attitude towards digital health services?**		
	Positive	118 (35)
	Rather positive	115 (34)
	Neutral	77 (23)
	Rather negative	17 (5)
	Negative	4 (1)
	No data	6 (2)
**Did you change your attitude due to the COVID-19 pandemic?**		
	Yes, the attitude has become more positive	135 (40)
	Yes, the attitude has become more negative	8 (2)
	No	185 (55)
	No data	9 (3)

### Use of DHTs

Almost half of the patients (143/337, 42%) were already using email for communication with physicians before the COVID-19 pandemic (Table 3). Currently, this value has increased to 58% (195/337). The number of patients using video consultations was very low: 4% (15/337). However, 14% (48/337) stated that they would use video consultations in the future. We found that 9% (32/337) were using reimbursed digital health applications (German: Digitale Gesundheitsanwendung; DiGA). Overall, 21% (71/337) were using other mobile health apps. Only 1% (3/337) had used blood self-sampling. Almost a quarter (78/337, 23%) stated that they wanted to use electronic prescriptions in the future.

**Table 3:** Digital health technology use.

	Used before COVID-19, n (%)	I currently use, n (%)	I will use in the future, n (%)	I am not interested, n (%)	I don’t use, n (%)	I don’t know, n (%)
Video consultation	7 (2)	15 (4)	48 (14)	14 (4)	191 (57)	53 (16)
Digital therapeutics (DiGA^a^)	14 (4)	32 (9)	38 (11)	10 (3)	154 (46)	86 (26)
mHealth apps (not DiGA)	19 (6)	71 (21)	40 (12)	10 (3)	152 (45)	54 (16)
Wearables	19 (6)	72 (21)	21 (6)	19 (6)	175 (52)	43 (13)
Blood self-sampling at home	2 (1)	3 (1)	9 (3)	50 (15)	187 (55)	99 (29)
Electronic prescription	7 (2)	30 (9)	78 (23)	5 (1)	147 (44)	75 (22)

^a^DiGA: digital health application.

### Potential Benefits of and Barriers to DHTs

Independence in terms of location (244/337, 72%) and time (216/337, 64%) and generally increased flexibility (170/337, 50%) were cited most often by patients as potential benefits of digital health. The detailed results are displayed in Table 4.

The biggest barrier at present is the lack of information. Thus, 49% (165/337) stated that they do not have sufficient knowledge, and 40% (134/337) stated that they are not sufficiently informed about DHTs. A third (118/337, 35%) stated that technical equipment is a barrier to using the tools.

**Table 4:** Potential benefits and barriers of digital health technologies.

Items		Participants (N=337), n (%)
**Potential benefits**		
	Location independent use	244 (72)
	Time-independent use	216 (64)
	Detailed documentation of the course of the disease	139 (41)
	Cost savings	134 (40)
	More options for accessing information, diagnostics, and therapy	125 (37)
	Accessibility	85 (25)
	More flexibility	170 (50)
	Better preparation for the physician-patient consultation	139 (41)
	Needs-based care	68 (20)
	No potential benefits	32 (9)
**Barriers**		
	Limited information about digital health services	134 (40)
	Insufficient evidence of the benefits of the offers	39 (12)
	Poor quality of current offers	32 (9)
	Gaps in data protection	92 (27)
	Lack of user-friendliness	84 (25)
	Lack of accessibility	12 (4)
	High costs	12 (4)
	Lack of technical equipment (eg, poor Internet connection, old end devices)	118 (35)
	Lack of knowledge among users	165 (49)
	No need because satisfied with the current analogue solutions	57 (17)

### Correlation Analyses

While correlation analysis revealed some relationships between content items (Table 5), no significant correlations between the content items and sociodemographic data were observed.

**Table 5:** Results of the correlation analyses.

Content items				Spearman ρ		*P* values (2-tailed)	
**Current use**							
	Positive attitude			0.458		<.001	
**Number of months diagnosed**							
	Number of months in rheumatology treatment			0.777		<.001	
**Potential benefits**							
	**Location-independent use**						
		Detailed documentation of disease progression	0.355		<.001		
		Accessibility	0.328		<.001		
		More flexibility	0.331		<.001		
		No	–0.525		<.001		
	**Detailed documentation of disease progression**						
		More options for accessing information, diagnostics, and therapy	0.330		<.001		
		More flexibility	0.336		<.001		
		Better preparation for the doctor-patient consultation	0.400		<.001		
		Needs-based care	0.330		<.001		
	**Cost savings**						
		Accessibility	0.380		<.001		
		More flexibility	0.320		<.001		
		Needs-based care	0.302		<.001		
	**More possibilities to access information, diagnostics, therapy**						
		Accessibility	0.346		<.001		
		More flexibility	0.405		<.001		
		Better preparation for the doctor-patient consultation	0.305		<.001		
	**Accessibility**						
		More flexibility	0.357		<.001		
		Needs-based care	0.338		<.001		
	**More flexibility**						
		Needs-based care	0.380		<.001		
		None	–0.307		<.001		
	**Better preparation for the doctor-patient consultation**						
		Needs-based care	0.360		<.001		
**Barriers**							
	Lack of technical equipment						
		Lack of knowledge among users	0.307		<.001		

## Discussion

To explore the current patient acceptance, use, and preferences for DHTs in rheumatology care, we performed a paper-pencil questionnaire survey among patients with RMD in Germany.

### Principal Findings

More than half (179/337, 53%) of 337 patients reported that they use DHTs. Overall, 21% (72/337) used wearables, 21% (71/337) used mHealth apps, 9% (32/337) used digital therapeutics (DiGA), 9% (30/337) used electronic prescriptions, 4% (15/337) used video consultations, and 1% (3/337) used at-home blood self-sampling. Nearly two-thirds of the patients with RMD (220/337, 65%) rated DHTs as useful. While 69% (233/337) reported a generally positive attitude toward DHTs, about 40% (135/337) mentioned their attitudes became more positive due to the COVID-19 pandemic. The main potential benefits of DHTs reported by the patients were location-independent use (244/337, 72%) and time-independent use (216/337, 64%). The main barriers included insufficient knowledge among users (165/337, 49%) and limited information about digital health services (134/337, 40%).

### Comparison With Previous Work

Our findings are aligned with previous studies that have examined patients’ acceptance of DHTs in rheumatology [3,14-19]. Comparing our results with previous findings, clear trends emerge that further underscore the growing acceptance of DHTs among patients with RMD.

In a survey conducted from September 2019 to December 2019 among 766 German patients with RMD [18], only 51% (364/718) of participants indicated familiarity with the term “telemedicine.” A mere 30% (210/690) expressed intentions to try telemedicine in the future, and a total of 21% (139/663) expressed a desire for their rheumatologist to offer telemedicine. In this study, conducted from February 2023 to April 2023, that is, after the COVID-19 period, 53% (179/337) of participants reported using DHTs. It should be noted, however, that while the terms telemedicine and DHTs are closely related, they are not synonymous.

In the 2019 survey, 0.3% of patients with RMD reported having experienced a video consultation with a physician. The current results reveal a 4% (15/337) use rate of video consultations, still representing a modest figure, suggesting that video consultations in rheumatology care remain an exceptional practice. These findings corroborate the outcomes observed by Richter et al [17]. In a survey of rheumatologists in Germany, 27% (55/205) reported offering video consultations during COVID-19 lockdown phases, with the frequency of provided video conferences diminishing as the pandemic progressed.

Knitza et al [15] reported that in 2018/2019, most patients with RMD (68%) believed that using medical apps could be beneficial for their own health [15]. However, out of 193 patients, only 8 (4%) were currently using medical apps. In the fall of 2020, Kernder et al [3] explored patient and rheumatologist attitudes toward digital technologies, particularly digital health applications. Even a higher rate of patients (222/299, 74%) and also rheumatologists (98/129, 76%) believed that digital health apps were valuable for managing RMDs. Compared with Knitza et al [15], our results reveal that digital health app use increased notably (71/337, 21%). In our study, a distinction was made between certified and prescribed digital therapeutics (DiGA) and other nonreimbursed digital health apps. Interestingly, patients reported less use of prescribed DiGAs (32/337, 9%) compared with other noncertified mHealth apps (21%). This discrepancy could be attributed to the absence of DiGAs explicitly tailored for RMDs, whereas non-DiGA mHealth apps fill this gap. A first pilot study evaluating DiGA use in rheumatology [20] revealed high patient acceptance and some clinical benefits, yet poor adherence as a major limiting barrier.

According to Kernder et al [3], 38% (112/299) of patients reported a positive change in attitude due to COVID-19, comparable to 40% (135/337) in this study. The most commonly cited advantages of DHAs were their independence from time and place, which were also expressed by participants in our study.

Our study also inquired about the use and acceptance of blood self-sampling, a prospect that has recently gained significance in future rheumatological care [11-13]. However, our results suggest that self-sampling currently remains largely confined to research settings.

In a recent secondary data analysis [21], we demonstrated that, specifically, older patients with RMD residing in rural areas, who could potentially benefit from telemedicine, currently lack the motivation to embrace it and appear to require additional support. However, these relationships were not confirmed by this study. Nevertheless, our data reaffirm the profound relevance of knowledge in the use of digital technologies in rheumatology care. In line with earlier findings [3,14,19,21], our participants identified “lack of knowledge among users” as the main barrier to use. Besides the patient perspective, the viewpoint of HCPs is also pertinent to the implementation of digital technologies. Lack of knowledge among rheumatologists was previously identified as a major barrier to implementing ePROs in routine care [22]. Conveniently, recent surveys also indicated a positive inclination of HCPs toward digital technologies in rheumatology [3,14,16,19].

### Limitations

Our study has several limitations. Despite our best efforts, it is possible that our survey did not capture all important emerging technologies relevant to rheumatology. In addition, the terminology used in this survey for DHTs may have caused confusion among participants, potentially leading to information bias. In addition, our survey reached a selected population from 3 outpatient clinics. This also applies to the selection of study participants, as we assume that individuals with a specific interest in digital health were more likely to have completed our questionnaire. A strength of previous surveys [3,16,17,19,22] is that the survey was paper-pen–based (instead of digital) to minimize selection bias.

Finally, it is essential to acknowledge that numerous research groups are focusing on measuring the use and acceptance in the domain of digital rheumatology. However, these studies often use varying approaches and terminologies and refer to different study populations. Therefore, the comparability of data across studies for the purpose of continuous monitoring of the digital transformation in rheumatology remains limited.

### Implications

Considering the heterogeneity regarding digital rheumatology surveys and fast transformation, we advocate for a standardized, regular, survey-based monitoring of the digital transformation in rheumatology from the perspectives of both patients with RMD and HCPs. Ideally, this monitoring should be conducted on an international level, including dedicated societies such as EULAR and the Digital Rheumatology Network. Furthermore, the specific digital technologies, their nomenclature, and other questionnaire content should be harmonized through Delphi studies in collaboration with an international expert board involving input from patients.

The results depict high acceptance regarding DHTs, which is currently limited mainly by a lack of knowledge. Dedicated education for patients with RMD and the treating HCPs is necessary to foster implementation in routine clinical practice.

### Conclusions

The digital transformation in rheumatology care in Germany is progressing. Patient acceptance and use is increasing. This provides hope that, despite the rising burden of disease and stagnating human resources, digital health can continue to ensure high-quality care for patients with RMD in the future. A prospective, standardized monitoring of digital transformation in rheumatology care is highly needed.

## Appendix

Multimedia Appendix 1 Original questionnaire (German).

Multimedia Appendix 2 Questionnaire - English translation.

## Abbreviations

DHT: digital health technology
DiGA: digital health application
ePRO: electronic patient-reported outcome
EULAR: European Alliance of Associations for Rheumatology
HCP: health care provider
mHealth: mobile health
RMD: rheumatic and musculoskeletal disease
