# Supplementary material for: Digital Transformation of Rheumatology Care in Germany: Cross-Sectional National Survey
Source: J Med Internet Res. 2025 Jan 6;27:e52601. doi: 10.2196/52601 (PMC11747535; doi:10.2196/52601)
Supplement: Multimedia Appendix 1 [file jmir_v27i1e52601_app1.pdf]

Im Folgenden verwenden wir die Begrifflichkeit der digitalen Gesundheitstechnologien. Darunter verstehen wir alle digitalen Mittel, die im Rahmen der ärztlichen Versorgung zum Einsatz kommen können: z.B. Videosprechstunden, Gesundheitsapps, Monitoringsysteme, Wearables.

1. Ich halte den Einsatz digitaler Gesundheitstechnologien in der rheumatologischen Versorgung für nützlich.

☐ Trifft gar nicht zu

☐ Trifft nicht zu

☐ Neutral

☐ Trifft zu

☐ Trifft voll zu

2. Wie sind Sie aktuell gegenüber digitalen Gesundheitstechnologien eingestellt?

☐ Positiv

☐ Eher positiv

☐ Neutral

☐ Eher negativ

☐ Negativ

3. Hat sich Ihre Einstellung gegenüber digitalen Gesundheitstechnologien seit der COVID-19 Pandemie verändert?

☐ Ja, sie ist positiver geworden

☐ Ja, sie ist negativer geworden

☐ Nein

4. Nutzen Sie aktuell digitale Gesundheitstechnologien?

☐ Ja

☐ Nein

Falls Sie <u>keine</u> digitalen Gesundheitstechnologien nutzen: Warum nutzen Sie <u>keine</u> digitalen Gesundheitstechnologien? (Mehrfachantworten möglich)

☐ Ich habe kein Interesse

☐ Persönliche Behandlungen können telemedizinisch nicht ergänzt werden

☐ Persönliche Behandlungen können telemedizinisch nicht ersetzt werden

☐ Ich habe Probleme mit der Netzabdeckung

☐ Ich habe Schwierigkeiten mit der Bedienung

5. Wie ist Ihr Nutzungsverhalten der folgenden Geräte oder Gesundheitstechnologien in der rheumatologischen Versorgung? Mehrfachauswahl möglich.

	Vor COVID-19 genutzt	Nutze ich aktuell	Werde ich in Zukunft nutzen	Daran habe ich kein Interesse	Nutze ich nicht	Kenne ich nicht
E-Mail	O	O	O	O	O	O
Videosprechstunde	O	O	O	O	O	O
Digitale Gesundheits-anwendungen (DiGA - Verschreibungsfähige mobile Apps oder Webanwendungen mit einem medizinischen Zweck)	O	O	O	O	O	O
Andere mobile Apps für Ihre Gesundheitsversorgung	O	O	O	O	O	O
Wearables (z.B. Smartwatch)	O	O	O	O	O	O
Selbstständige Blutentnahme zu Hause	O	O	O	O	O	O
e-Rezept	O	O	O	O	O	O

6. Welche Vorteile sehen Sie beim Einsatz digitaler Gesundheitstechnologien (bspw. Videosprechstunden, Gesundheitsapps, Monitoringsystemen, Wearables)? Mehrfachauswahl möglich.

☐ Ortsunabhängige Nutzung
☐ Zeitlich unabhängige Nutzung
☐ Detaillierte Dokumentation des Krankheitsverlaufs
☐ Kostenersparnis
☐ Mehr Möglichkeiten, um an Informationen, Diagnostik und Therapie zu gelangen
☐ Barrierefreiheit
☐ Mehr Flexibilität
☐ Bessere Vorbereitung auf das Arzt-Patienten-Gespräch
☐ Bedarfsgerechte Versorgung
☐ Keine

7. Welche Barrieren sehen Sie beim Einsatz digitaler Gesundheitstechnologien (bspw. Videosprechstunden, Gesundheitsapps, Monitoringsystemen, Wearables)? Mehrfachauswahl möglich.

☐ Wenig Informationen über Angebote (durch Ärzt:innen, Krankenkassen etc.)

☐ Zu wenige Belege für den Nutzen der Angebote

☐ Schlechte Qualität der aktuellen Angebote

☐ Lücken im Datenschutz

☐ Mangelnde Benutzerfreundlichkeit

☐ Mangelnde Barrierefreiheit

☐ Hohe Kosten

☐ Fehlende technische Ausstattung (schlechte Internetverbindung, alte Endgeräte, etc.)

☐ Fehlendes Wissen bei den Anwender:innen

☐ Kein Bedarf, da zufrieden mit den aktuellen analogen Lösungen

Im Folgenden stellen wir Ihnen einige soziodemographische Fragen. Die Angaben helfen, die Ergebnisse dieser Umfrage zu untersuchen bzw. auszuwerten. Wir möchten Sie hier nochmals darauf hinweisen, dass die Befragung anonym ist, d.h. dass keine Rückschlüsse auf Ihre Person gemacht werden können.

8. Seit wann sind Sie in rheumatologischer Behandlung?

_ _ . _ _ . _ _ _ _
(Tag) (Monat) (Jahr)

9. Welche Diagnose wurde bei Ihnen gestellt?

☐ Rheumatoide Arthritis

☐ Systemischer Lupus Erythematodes

☐ Psoriasis Arthritis

☐ Spondyloarthritis

☐ Axiale Spondyloarthritis

☐ Sjörgen Syndrom

☐ Fybromyalgie

☐ Andere: ________________________

10. Wann wurde die Diagnose gestellt? Bitte geben Sie die Jahreszahl an.

_ _ _ _
(Jahr)

11. Wie alt sind Sie?

________ Jahre

12. Zu welchem Geschlecht ordnen Sie sich zu?

☐ Weiblich
☐ Männlich
☐ Divers

13. Was ist Ihr höchster allgemeinbildender Schulabschluss?

☐ Ich bin noch Schüler/-in
☐ Von der Schule abgegangen ohne Schulabschluss

☐ Hauptschulabschluss (Volksschulabschluss) oder gleichwertiger Abschluss

☐ Realschulabschluss (Mittlere Reife) oder gleichwertiger Abschluss

☐ Abitur, fachgebundene Hochschulreife oder gleichwertiger Abschluss

☐ Einen anderen Schulabschluss, und zwar: ___________________________________

14. Was ist Ihr höchster beruflicher Ausbildungsstatus?

☐ Noch in beruflicher Ausbildung

☐ Ohne beruflichen Ausbildungsabschluss

☐ Abschluss einer beruflichen Ausbildung von mindestens einem Jahr

☐ Hochschulabschluss

15. Bitte geben Sie an, wie groß der Ort ist, in dem Sie wohnen.

☐ Ländliche Region (Gemeinde unter 5.000 Einwohner)

☐ Kleinstadt (5.000 – 20.000 Einwohner)

☐ Mittelstadt (20.000 – 100.000 Einwohner)

☐ Großstadt (über 100.000 Einwohner)

☐ Millionenstadt (über 1.000.000 Einwohner)

16. Bitte geben Sie die ersten <u>drei</u> Ziffern Ihrer Postleitzahl an: ____________

- Vielen Dank für Ihre Teilnahme -
