# Supplementary material for: Digital Transformation of Rheumatology Care in Germany: Cross-Sectional National Survey
Source: J Med Internet Res. 2025 Jan 6;27:e52601. doi: 10.2196/52601 (PMC11747535; doi:10.2196/52601)
Supplement: Multimedia Appendix 2 [file jmir_v27i1e52601_app2.pdf]

Below, we use the term digital health technologies. We understand this to include all digital means that can be used in medical care, e.g., video consultations, health apps, monitoring systems, wearables.

1.Digital health technologies are useful.

☐ Strongly disagree
☐ Disagree
☐ Neutral
☐ Agree
☐ Strongly agree

2. How do you rate your attitude towards digital health technologies?
☐ Positive
☐ Rather positive
☐ Neutral
☐ Rather negative
☐ Negativ

3. Did you change your attitude due to COVID-19 pandemic?
☐ Yes, more positive.
☐ Yes, more negative.
☐ No

4. Do you use digital health technologies?
☐ Yes
☐ No

If you do not use digital health technologies: Why do you not use digital health technologies? (Multiple answers possible)
☐ I am not interested.
☐ Personal treatments cannot be supplemented by telemedicine.
☐ Personal treatments cannot be replaced by telemedicine.
☐ I have problems with network coverage.
☐ I have difficulties using digital health technologies.

5. What is your usage behavior with the following devices or health technologies in rheumatology care? (Multiple answers possible)

	Used before COVID-19	I am currently using	I will use in the future	I am not interested	I do not use	I do not know
E-Mail	O	O	O	O	O	O
Video consultation	O	O	O	O	O	O
Digital Health Applications (DiGA - Prescribable mobile apps or web applications with a medical purpose)	O	O	O	O	O	O
Other mobile apps for your health care	O	O	O	O	O	O
Wearables (e.g. Smartwatch)	O	O	O	O	O	O
Self-administered blood sampling at home	O	O	O	O	O	O
e-Prescription	O	O	O	O	O	O

6. What facilitators do you see in using digital health technologies (e.g., video consultations, health apps, monitoring systems, wearables)? (Multiple answers possible)

☐ Location-independent use
☐ Time-independent use
☐ Detailed documentation
☐ Cost savings
☐ Access to information, diagnostics & therapy
☐ Accessibility
☐ More Flexibility
☐ Better preparation for doctor-patient conversations
☐ Needs based care
☐ None

7. What barriers do you see in using digital health technologies (e.g., video consultations, health apps, monitoring systems, wearables)? (Multiple answers possible)

☐ Limited information about digital health technologies

☐ Insufficient evidence

☐ Poor quality of current offers

☐ Gaps in data protection

☐ Lack of user-friendliness

☐ Lack of accessibility

☐ High costs

☐ Lack of technical equipment

☐ Lack of knowledge among users

☐ No need / satisfied with current care

In the following, we ask you some sociodemographic questions. The information helps to analyze the results of this survey. Please note again that the survey is anonymous, meaning no conclusions can be drawn about your identity.

8. Since when have you been receiving rheumatologic treatment?

_ _ . _ _ . _ _ _ _ _

(Day) (Month) (Year)

9. What diagnosis have you been given?

☐ Rheumatoid Arthritis

☐ Systemic Lupus Erythematodes

☐ Psoriatic Arthritis

☐ Spondyloarthritis

☐ Axial spondyloarthritis

☐ Sjörgen Syndrom

☐ Fibromyalgia

☐ Other ________________________

10. When was your diagnosis given? Please provide the year.

_ _ _ _

(Year)

11. How old are you?

________ Years

12. What gender do you identify with?

☐ Female
☐ Male
☐ Diverse

13. What is your highest level of general education?

☐ I am still a student.
☐ Left school without a diploma.

☐ Basic school leaving certificate

☐ Intermediate school leaving certificate

☐ Advanced school leaving certificate

☐ Another type of school leaving certificate, namely:
____

14. What is your highest vocational training status?

☐ Currently in vocational training

☐ Without a vocational qualification

☐ Completed vocational training of at least one year

☐ University degree

15. Please indicate the size of the place where you live.

☐ Rural area (community with less than 5,000 inhabitants)

☐ Small town (5,000 – 20,000 inhabitants)

☐ Medium-sized town (20,000 – 100,000 inhabitants)

☐ Large city (over 100,000 inhabitants)

☐ Metropolis (over 1.000.000 Einwohner)

16. Please provide the first three digits of your postal code:: ____________

- Thank you for your participation -
